# Development and In Vitro Characterization of Antibiotic-Loaded Nanocarriers for Dental Delivery

**DOI:** 10.3390/molecules28072914

**Published:** 2023-03-24

**Authors:** Mohammed Ghazwani, Rajalakshimi Vasudevan, Geetha Kandasamy, Umme Hani, Gaddam Niharika, Manusri Naredla, Praveen Devanandan, Ranadheer Chowdary Puvvada, Abdulrahman A. Almehizia, Abdulrahim R. Hakami, Rajeshri Dhurke

**Affiliations:** 1 Department of Pharmaceutics, College of Pharmacy, King Khalid University (KKU), Abha 61421, Saudi Arabia; 2 Department of Pharmacology, College of Pharmacy, King Khalid University (KKU), Abha 61421, Saudi Arabia; 3 Department of Clinical Pharmacy, College of Pharmacy, King Khalid University (KKU), Abha 61421, Saudi Arabia; 4 Department of Pharmaceutics, St. Peter’s Institute of Pharmaceutical Sciences, Hanamkonda 506001, Telangana, India; 5 Department of Pharmacy Practice, St. Peter’s Institute of Pharmaceutical Sciences, Hanamkonda 506001, Telangana, India; 6 Department of Pharmaceutical Chemistry, College of Pharmacy, King Saud University, P.O. Box 2457, Riyadh 11451, Saudi Arabia; 7 Department of Clinical Laboratory Sciences, College of Applied Medical Sciences, King Khalid University (KKU), Abha 61481, Saudi Arabia

**Keywords:** nanocarriers, β tricalcium phosphate, periodontitis, ciprofloxacin hydrochloride

## Abstract

The aim of this research work was to formulate and evaluate ciprofloxacin hydrochloride-loaded nanocarriers for treating dental infections and bone regeneration. Periodontal infection is associated with inflammation, soft tissue destruction, and bone loss. The objective of the study was to extract β tricalcium phosphate (β-TCP) from coral beach sand using the hydrothermal conversion method and load these nanocarriers with ciprofloxacin hydrochloride. The developed drug-loaded nanocarriers were evaluated for various parameters. In vitro drug-loading studies showed the highest drug loading of 71% for F1 with a drug: carrier ratio compared to plain ciprofloxacin hydrochloride gel. β-TCP and nanocarriers were evaluated for powder characteristics and the results were found to have excellent and fair flowability. In vitro drug release studies conducted over a period of 5 days confirmed the percentage drug release of 96% at the end of 120 h. Nanocarriers were found to be effective against *S. aureus* and *E. coli* showing statistically significant antibacterial activity at (* *p* < 0.05) significant level as compared to plain ciprofloxacin hydrochloride gel. The particle size of β-TCP and nanocarriers was found to be 2 µm. Fourier transform infra-red studies showed good compatibility between the drug and the excipients. Differential scanning calorimetry studies revealed the amorphous nature of the nanocarriers as evident from the peak shift. It is obvious from the XRD studies that the phase intensity was reduced, which demonstrates a decrease in crystallinity. Nanocarriers released the drug in a controlled manner, hence may prove to be a better option to treat dental caries as compared to conventional treatments.

## 1. Introduction

Periodontitis is a chronic inflammatory disease that affects the periodontal ligament, gingiva, and alveolar bone, which are the main structural support of the tooth. Periodontitis is a complex infectious disease that causes both host- and bacteria-mediated tissue death and requires the administration of numerous medications [1,2]. Periodontitis is mostly caused by bacterial infection. The host-mediated reactions set off by the bacterial enzymes degrade the extracellular matrix, causing inflammation and bone loss. As a result, developing local drug delivery systems is preferable [3,4].

Conventional treatments for periodontitis aim to reduce inflammation and infection sources to prevent the progression of the disease but this treatment does not achieve the regeneration of tissues that have been lost due to infection [5]. Over the past few years, nanomaterials and regenerative periodontal therapies such as tissue engineering, bone grafting, nanomaterials’ scaffold, nanocarriers, etc., have gained importance in dental applications [6,7,8]. These drug delivery approaches provide a suitable path through which drugs incorporated into nanocarriers or scaffolds can be targeted at the inflamed periodontal site.

Bone fillers are used locally to treat the bone abnormalities caused by the infection’s concomitant bone loss. It would be best to administer various medications locally because systemic administration is associated with side effects and inadequate concentration at the site of infection, which might cause diversity in the clinical response [9]. Local drug delivery systems offer several benefits, including a high concentration of drugs at the target site, an extended duration of therapeutic action, and fewer side effects, all of which contribute to more efficient and safe treatment [10].

Calcium phosphate ceramics are suitable candidates for drug administration to the bone; some of the Calcium Phosphate Ceramics (CPC) that are frequently used for biomedical applications include hydroxyapatite, calcium-deficient hydroxyapatite, and β-tricalcium phosphate (β-TCP) [11]. The local drug-delivery system based on CPC offers the additional benefit of bone regeneration and repair, which is important for the treatment of periodontitis.

Ciprofloxacin (CIP) is a broad-spectrum fluoroquinolone that is widely used for the treatment of multiple bacterial infections. It has the advantage of having a low minimum inhibitory concentration for *S. aureus* and is effective against both gram-positive and gram-negative bacteria [12]. Ciprofloxacin hydrochloride has been extensively studied in the form of nanofibers and nanocomposites for dental applications. Antibiotics serve as an effective treatment for this condition as it is difficult to access periodontopathic organisms that are deeply rooted in the periodontal pocket and those that are invasive in nature [13,14]. Local delivery of antibiotics would help overcome the limitations of systemic antimicrobial therapy and has gained remarkable interest. Antibiotics are matrixed and immobilized on the micro- and nanomaterials for improved stability, localized delivery, and sustained release [15,16].

Coral is a natural structure having optimal strength and structural characteristics that are compatible with those of bone. Corals, including those of Indian and Australian origin, have been successfully converted to coralline hydroxyapatite and tricalcium phosphate [17,18]. Natural coral implanted into the bony tissue is gradually resorbed and replaced by newly formed bone. Coral has been used clinically with good results in spinal fusion or to fill periodontal defects [19]. 

Coral sand has an edge over various other materials used for bone healing, primarily due to its high calcium carbonate content that has exhibited osteoconductive, biocompatible, and biodegradable potential. Although it is incapable of being osteoinductive, it has shown to facilitate cell attachment and bone healing by differentiation acting as a channel to carry growth factors. Its bone grafting potential has been remarkable when the bone formation rate at the site of implantation is adjusted on par with the rate of resorption [20].

The aim of the present work was to use β-TCP as the carrier material for loading ciprofloxacin hydrochloride, which is a frequently used antibiotic for treating bone infection. Hence, an attempt was made to load ciprofloxacin hydrochloride in β-TCP that was extracted from coral sand in the form of a nanocarrier. This developed ciprofloxacin hydrochloride-loaded nanocarrier would be able to release antibiotics for a longer period of time, stop and prevent the further progress of the infection, and also initiate regeneration of the bone and bone repair by faster reabsorption in the bone.

## 2. Results

### 2.1. Characterization

Identification tests for beta-tricalcium phosphate

Identification test for phosphate

The identification test for phosphate shows the formation of a yellow precipitate, which confirms the presence of phosphate in the sample. 

Identification test for calcium

The calcium identification test shows white precipitate and ash formation in the flame test, which confirms the presence of calcium. The above two identification tests confirm the presence of phosphate and calcium in synthesized β-TCP, as given in Table 1.

Micromeritic properties: Micromeritic properties such as angle of repose, Hausner ratio, and Carr’s index were performed to determine the flow properties From the above data θ values were well within the limit and indicating reasonably good flow potentials for the prepared nanocarriers. The values of Carr’s index for formulation F1 was found to be 115.55% It shows that nanocarriers contain good flow characteristics The values of Tapped density for ranged between 0.78 g/cm^3^ respectively. Density difference between the formulations is negligible and the density values of the formulations were well within the limit, indicating the prepared nanocarriers were non aggregated and spherical in nature compared to the other formulations. The results demonstrate that nanocarriers’ formulation F1 showed good and fair flowability compared to other formulations as observed in Table 2.

### 2.2. In Vitro Drug-Loading Studies

**Figure 1:**
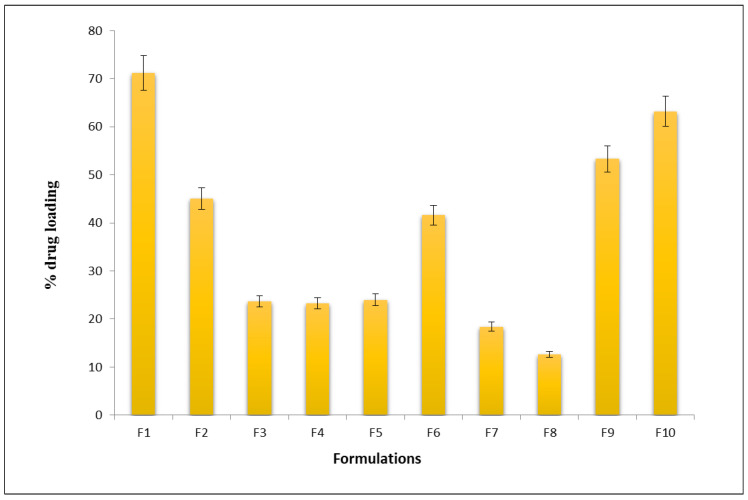
In vitro drug-loading profile of the nanocarriers formulation (bars represent mean ± SD; *n* = 6).

In vitro drug-loading studies performed on developed nanocarrier formulations showed the highest amount of drug loading of about 71% for the F1 formulation consisting of (1:1, drug: carrier) compared to other formulations, which showed only 10–40% of drug loading as shown in Figure 1. Based on the results obtained from the drug loading studies, the F1 formulation was selected as the best formulation for further studies. Drug loading for plain ciprofloxacin hydrochloride gel was found to be 100% as the drug was dispersed in the carbopol base gel.

### 2.3. Drug Content of Plain Gel

The drug content of the plain gel loaded with ciprofloxacin hydrochloride was found to be 99.12 ± 0.23 which was within the limits.

### 2.4. In Vitro Drug Release

**Figure 2:**
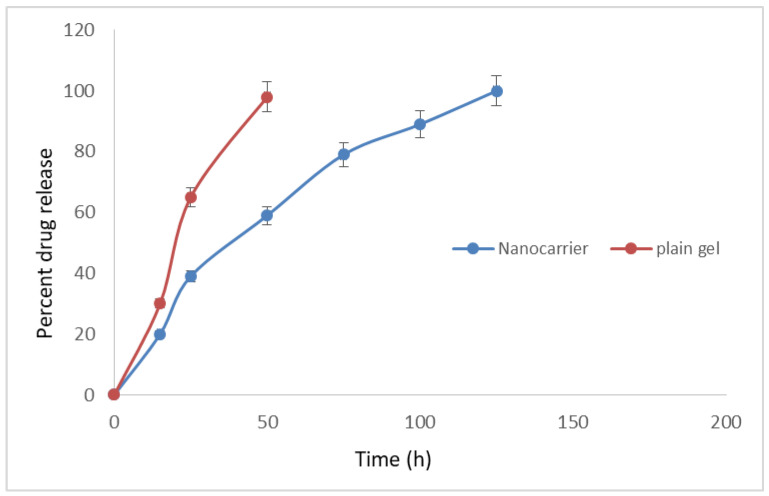
In vitro drug-release profile of the nanocarrier formulation (F1) and the plain gel loaded with ciprofloxacin hydrochloride (bars represent mean ± SD; *n* = 6).

From Figure 2, it is evident that the F1 formulation showed the highest amount of drug release of 96 ± 0.35% at the end of 120 h. The drug was released in a controlled manner over a period of 5 days which may prove beneficial for showing a local effect in dental caries. Plain gel loaded with ciprofloxacin hydrochloride released 100% of the drug within 50 h.

### 2.5. Anti-Bacterial Studies

**Figure 3:**
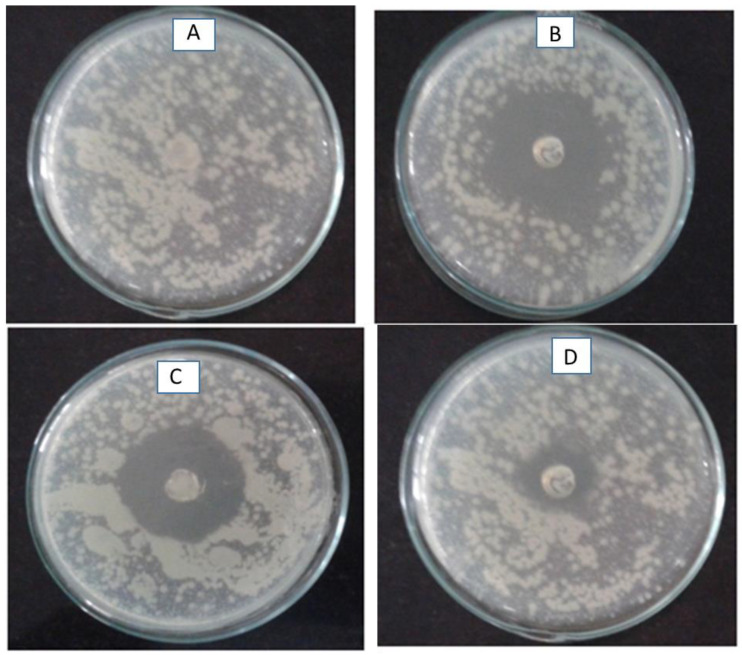
Anti-microbial studies of (**A**) Placebo (**B**) Nanocarrier formulation (F1) in *Staphylococcus aureus* species (**C**) Nanocarrier formulation (F1) in *Escherichia coli* species. (**D**) Plain gel loaded with ciprofloxacin hydrochloride.

Formulation F1 was further evaluated for antimicrobial activity against *Staphylococcus aureus* and *Escherichia coli* by the agar diffusion method. The zone of inhibition is shown in Figure 3. The highest zone of inhibition of 3.5 cm was observed for *Staphylococcus aureus*, 3 cm for *E. coli*, while the control did not show the zone of inhibition. The zone of inhibition for plain gel loaded with ciprofloxacin hydrochloride showed a zone of inhibition of 1.5 cm. It is evident from the antimicrobial studies that nanocarriers were effective against both species, showing slightly high activity for the *Staphylococcus aureus* species.

### 2.6. Scanning Electron Microscopy

**Figure 4:**
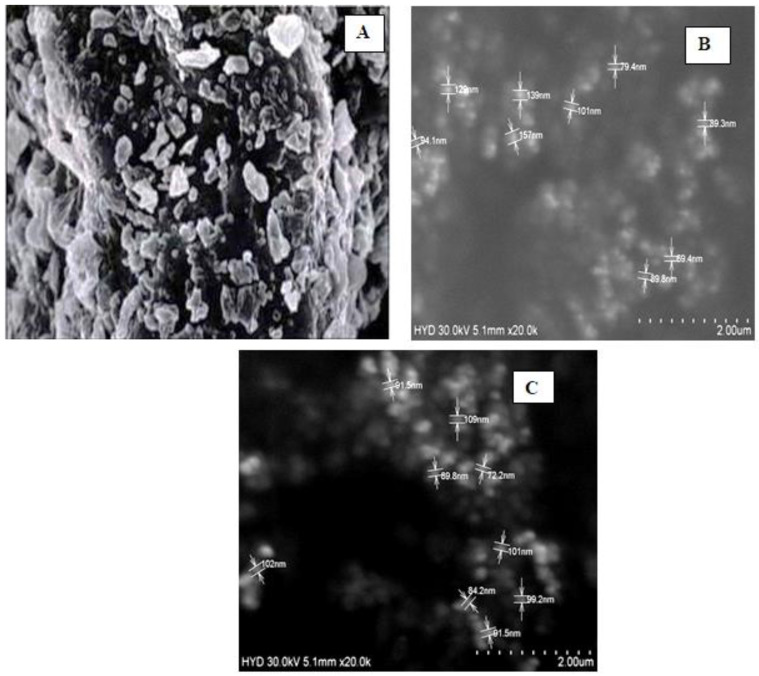
SEM images of (**A**) Ciprofloxacin hydrochloride (**B**) β-TCP (**C**) Nanocarriers’ formulation.

The SEM image Figure 4A of pure ciprofloxacin shows irregular shape particles with a rough surface with a size range of 2 ± 0.15 μm. From the measurements given on the SEM images, it is evident that β-TCP (B) has a particle size in the nanometer range from 89 nm to 155 nm with a particle having a spherical shape with a smooth surface. The SEM image of a nanocarrier loaded with ciprofloxacin (C) showed that the particle sizes in the nanometer range, i.e., from 89 to 110 nm, had a smooth surface.

### 2.7. Differential Scanning Calorimetry (DSC)

**Figure 5:**
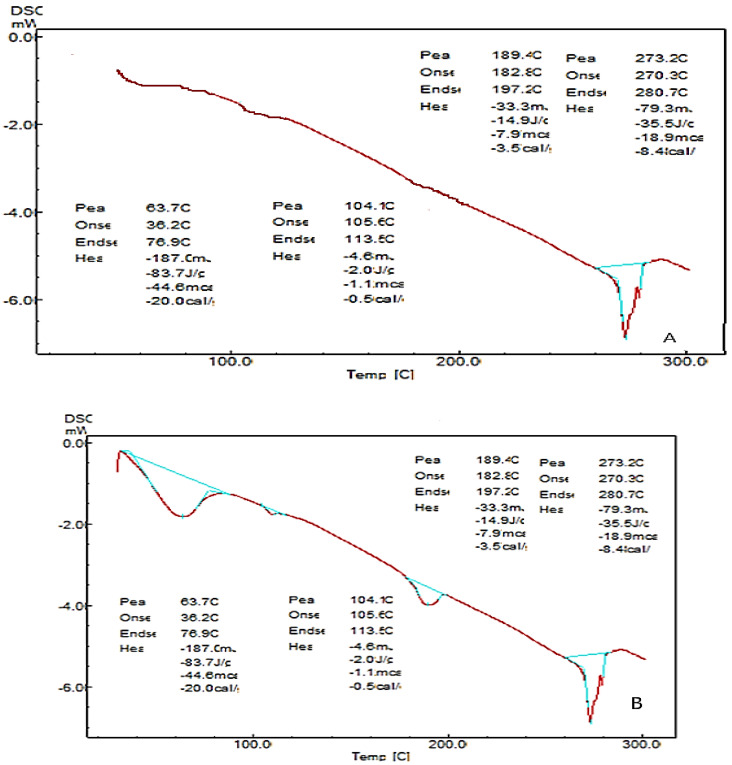
DSC thermograms of (**A**) pure drug and (**B**) nanocarrier.

The thermotropic behavior and the physical state of the drug in nanocarrier formulation were evaluated by performing DSC analysis. In Figure 5, it is observed that the drug shows a sharp endothermic peak at 280 °C with an enthalpy of 80 J/g which represents the melting point of the pure drug. β-TCP shows an endothermic peak at 193.5 °C with an enthalpy of 137.8 J/g. The nanocarriers’ formulation shows endothermic peaks at 63 °C, 104 °C, and 280.41 °C with an enthalpy of 83 J/g, 2 J/g, and 14 J/g. The result indicates that there is no drug excipient incompatibility, as there is no change in the drug peak. This confirms that the drug has been embedded in the matrix of β-TCP.

### 2.8. Fourier Transform Infrared Spectroscopy (FTIR)

The infrared spectra of pure drug and physical mixture were recorded in a range of 3100 cm^−1^–800 cm^−1^ using a FTIR spectrophotometer. 

**Figure 6:**
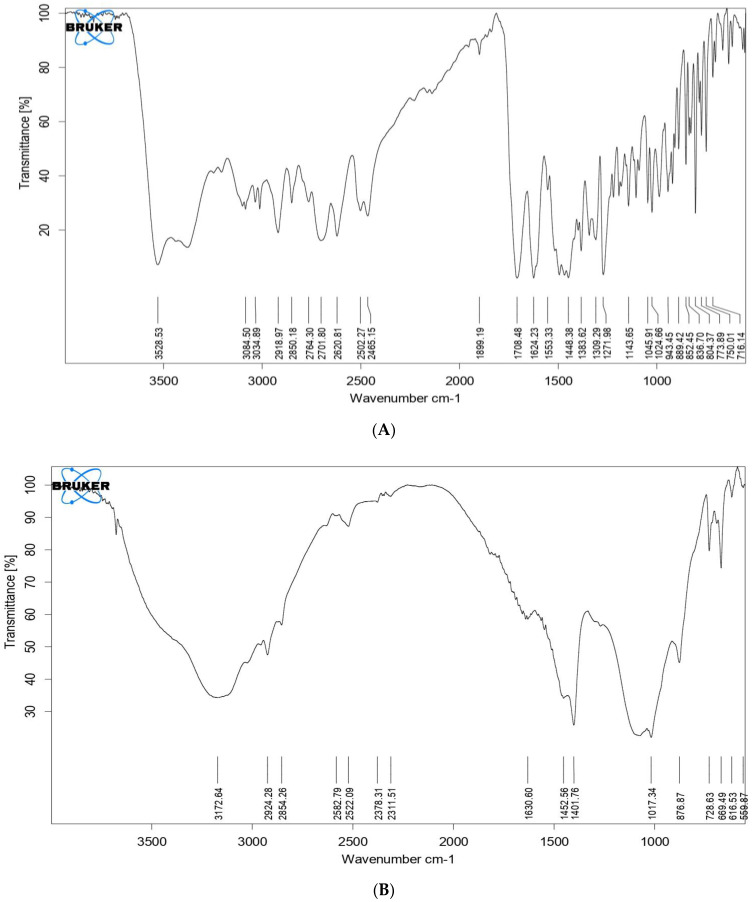

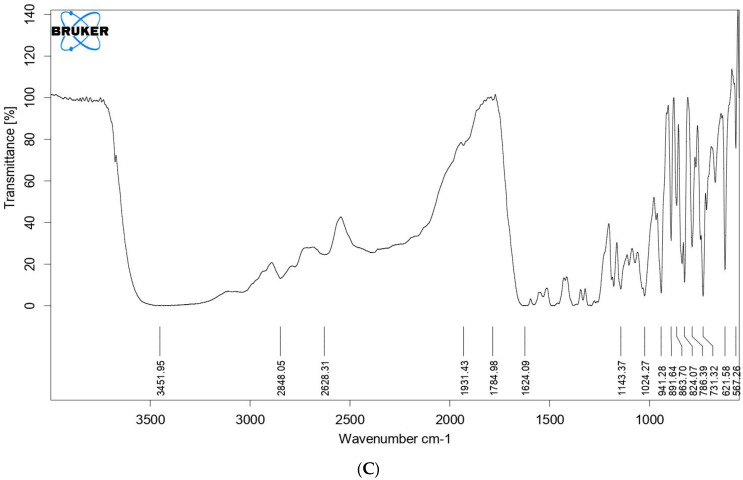
FTIR with characteristics peaks of (**A**) β-TCP (**B**) Pure drug (**C**) Nanocarriers’ formulation.

From Figure 6, it is evident that the individual spectra of pure drug, β-TCP, and formulation show characteristic peaks. for the drug band at 3084 cm^−1^ is assigned to stretching vibration of the amine group, and the band at 2620 cm^−1^ is assigned to the stretching vibration of the carboxylic acid group. The band at 773 cm^−1^, 1708 cm^−1^, and 836 cm^−1^ is due to the stretching vibration of the alkyl halide, ketone, and benzene groups, respectively. The characteristic peaks for ciprofloxacin hydrochloride in nanocarriers have the same wavenumber as that of spectra obtained for pure ciprofloxacin hydrochloride, there is no shift in the wave number which indicates that there is no incompatibility between the drug and the carrier.

### 2.9. XRD-X-ray Diffraction

**Figure 7:**
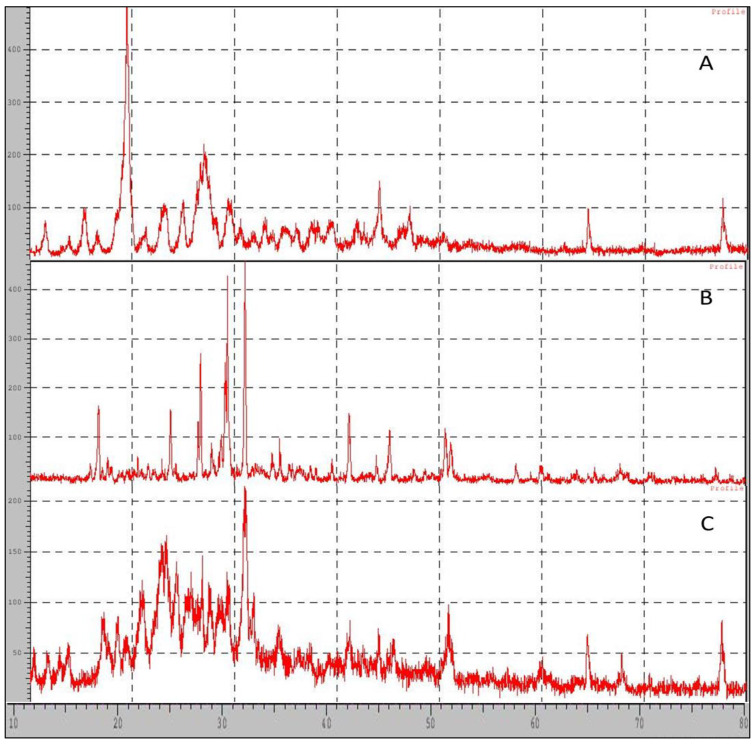
X-RD pattern of (**A**) Pure drug (**B**) β—TCP, (**C**) Nanocarriers (F1).

X-ray powder scattering measurements of the ciprofloxacin hydrochloride, nanocarriers, and beta-tricalcium phosphate were carried out to understand the crystallinity of the pure drug or any loss or modification of the crystallinity of the pure drug after its formulation. The pure drug generated three prominent peaks at 2ø of 19°, 27°, and 26.4° with an intensity of 286, 117, and 94, respectively, while nanocarriers generated the peak at 2ø of 30°, 23.26°, and 22.82° with an intensity of 101, 62, and 60, respectively. If the prominent peaks of drugs, for example, at 2ø of 19°, 27°, and 26.4°, are compared with the peak intensity obtained for nanocarrier they are 14, 27, and 33, respectively. The intensity of the peak was highly reduced, peaks are not prominent but rather broad which indicates modification in crystallinity due to the formation amorphous state, as seen in Figure 7. Furthermore, the particle size was calculated using the Scherrer formula, Dp=(0.94×λ)/(β×cos θ) where *Dp* = average crystallite size, *β* = line broadening in radius, *θ* = Bragg angle, and *λ* = X ray wavelength, and 0.94 is the Scherrer constant value. The calculated results obtained for particle size for β-TCP and nanocarrier was found to be 47.04 nm and 17.58 nm, respectively. 

## 3. Discussion

As discussed previously, periodontitis is a serious infection that causes inflammation of gums, periodontal ligaments, and dental cementum; in addition, if not treated, it may result in tooth loss. Conventional methods which are used for the treatment include scaling of the tooth and planning of the root canal along with the topical and systemic administration of antibiotics. These conventional treatments do not treat the disease as they have limited access to the affected site. Multiple doses of antibiotics are essential to counteract infection and also for preventing further infection. Antibiotic resistance to first-line agents has been reported due to the decline in antibiotic concentration to the subtherapeutic level. Ciprofloxacin hydrochloride, being a broad-spectrum antibiotic, serves as an ideal candidate as it has a low frequency of resistance against microbes. The objective of using ciprofloxacin hydrochloride in the present research was due to its high water solubility and high tissue permeability which allows it to reach the affected site easily. To increase its penetrability more and to provide controlled release over longer period of time ciprofloxacin was loaded into the nanocarrier system. This nanocarrier system will not only show greater penetrability but also help in bone regeneration.

In the present studies, -TCP extracted β-TCP from coral sand was used, as it exactly mimics the structure of human bone and is biocompatible and biodegradable. β-TCP from coral sand was extracted using the hydrothermal conversion method, as it retains the porosity and structure of the coral. As periodontitis leads to bacterial infection and bone loss, ciprofloxacin hydrochloride along with β-TCP were used. Converted β-TCP was tested for the presence of calcium and phosphate was tested by chemical identification test. Then, it was formulated into nano carriers. In vitro drug-loading studies were performed in which all formulations of the F1 (1:1) drug carrier showed 71% drug loading and 96 ± 0.35% of drug release at the end of 120 h compared to plain ciprofloxacin gel, which released the complete drug in just 50 h. Fast drug release may be due to two factors, the first one being ciprofloxacin dispersed in the carbopol base which resulted in a shorter diffusion path length for the drug to obtain release from the gel base. The second one would be due to the short residence time of the plain gel. Nanocarriers, on the other hand, provided a longer diffusion path, since the drug has to release from the matrix of β-TCP retarding the release in a controlled manner, which results in the controlled release of the drug over a period of 120 h.

The enhanced antimicrobial activity may be attributed to the increased diffusion of nanocarriers throughout the agar medium. Similarly, plain gel loaded with ciprofloxacin hydrochloride showed less activity, as the diffusion of the drug through the agar medium is low because of the larger size, which makes it difficult to diffuse in the agar medium. The one-way ANOVA followed by Bonferroni’s multiple comparison tests shows that formulation F1, i.e., ciprofloxacin nanocarriers, showed statistically significant antibacterial activity at (* *p <* 0.05) significant level (* *p* < 0.05). 

A study by Gupta et al. (2014) reported the development of a mucoadhesive gel containing chlorhexidine for local drug delivery. The formulation showed good mucoadhesive properties and was able to release the drug in a sustained manner over a period of six hours [21]. However, the formulation observed low drug-loading efficiency and moderate antimicrobial activity against *S. mutans*. 

Another study by Suresh et al. (2013) reported the development of a mucoadhesive gel containing ciprofloxacin for local drug delivery. The formulation was found to have good mucoadhesive properties and was able to release the drug in a sustained manner over a period of 24 h. However, the formulation showed low drug-loading efficiency and moderate antimicrobial activity against *S. mutans* [22].

In contrast, the current study reports a nanocarrier-based local drug-delivery system that has high drug-loading efficiency and is able to release the drug in a controlled manner over a period of five days. The formulation also demonstrates potent antimicrobial activity against both *S. aureus* and *E. coli*. These results indicate that the current formulation may have better efficacy in the treatment of dental caries compared to previous formulations. 

A study by Samiei et al. (2016) developed a nanofiber-based local drug-delivery system containing doxycycline for the treatment of periodontitis. The formulation was found to have good mechanical properties, sustained release of the drug for up to 10 days, and significant antimicrobial activity against *P. gingivalis* and *A. actinomycetemcomitans*. Although this study did not investigate the efficacy specifically for the treatment of dental caries, the sustained release and antimicrobial activity demonstrated by the formulation could potentially be beneficial for this indication as well [23].

Another study by Muthu et al. (2013) developed a nano gel-based local drug-delivery system containing ciprofloxacin for the treatment of periodontitis. The formulation was found to have sustained release of the drug for up to 24 h and significant antimicrobial activity against *P. gingivalis* and *A. actinomycetemcomitans*. However, the study did not investigate the efficacy specifically for the treatment of dental caries [24].

A study by Duan et al. (2021) developed a nanoemulsion-based local drug-delivery system containing cinnamaldehyde for the treatment of dental caries. The formulation was found to have sustained release of the drug for up to 8 h and significant antimicrobial activity against *S. mutans*. However, the study did not investigate the efficacy against other microorganisms commonly associated with dental caries [25].

Comparing these studies with the current study, it is clear that each formulation has its own unique advantages and disadvantages. The current formulation has high drug-loading efficiency and sustained release for up to five days, as well as potent antimicrobial activity against both *S. aureus* and *E. coli*. However, it is important to note that the studies mentioned above also demonstrate promising results and may be effective in the treatment of dental caries or related conditions such as periodontitis. Ultimately, the choice of the formulation may depend on factors such as the specific microorganisms involved in the disease, the desired duration of drug release, and the drug loading efficiency required.

Further from the scanning electron microscopy studies, it was evident that ciprofloxacin particle size was converted to nanosize within the matrix of β-TCP. DSC and FTIR studies revealed that there was no significant change in the chemical integrity of the drug and the functional groups present in the pure drug, carrier, and nanocarrier formulation. This indicates that there was no chemical interaction between the drug and the excipients. It can be concluded from DSC studies that ciprofloxacin has been embedded in the β-TCP which was further confirmed by XRD studies which shows that the intensity of the peak of the drug was highly reduced compared to the pure form of ciprofloxacin, so it can be stated that most of the crystalline form of ciprofloxacin has been converted to the amorphous form. Data obtained from the above research studies show promising results in vitro for dental nanocarriers consisting of ciprofloxacin hydrochloride loaded in β-TCP. 

The limitations of this study are that the authors were able to develop a nanocarrier-based delivery system for dental caries, which showed good in vitro results, but further in vivo studies are needed to determine the effectiveness of the formulation in dental caries and also to check its affinity in bone rejuvenation. The next step could involve conducting animal studies to evaluate the effectiveness of the developed nanocarrier for treating bone infections and promoting bone regeneration. This would involve assessing the biocompatibility and safety of the nanocarrier, as well as its ability to deliver the antibiotic to the site of infection. Future studies could focus on optimizing the drug delivery system by exploring different carrier materials, antibiotic dosages, and release mechanisms. This could involve using computational modeling and simulation techniques to design and optimize the drug delivery system.

## 4. Materials and Method

Ciprofloxacin hydrochloride and diammonium hydrogen phosphate were obtained from Sigma-Aldrich (CAS number: 86393-32-0) and (CAS number: 7783-28-0), respectively. Coral sand was purchased from eBay India, and ammonia and other chemicals were purchased from Finar Chemicals Hyderabad. All other chemicals and reagents used were of analytical grade.

### 4.1. Extraction of Beta Tricalcium Phosphate from Coral Beach Sand by Using Hydrothermal Conversion Method

Accurately weigh 225 gm of coral sand and immerse it in sodium hypochlorite solution for 20 min, dry it at 40 °C for 2 h to remove residual organics in a vacuum oven at 400 mm hg pressure. To this, add 125 gm of ammonium dihydrogen phosphate and keep it in a hot air oven at 225 °C for 48 h. The resulting product formed was dried at 100 °C for 2 h. Under a controlled hydrothermal exchange process, a mixed product of calcite and beta-tricalcium phosphate can be derived from coral sand grains with a porous structure [19,20,21]. A fine powder was then created using a mortar and pestle.

### 4.2. Preparation of Nanocarriers

Nanocarriers were prepared by taking different ratios of drug and carrier as given in the Table 3, to that phosphate buffer saline solution was added and then the mixture was allowed to settle for 24 h. After 24 h, the mixture was filtered, and the amount of drug loaded was determined by using a UV–Visible spectrophotometer at 275 nm. The precipitate was air-dried and used for further characterization. The optimized formulation was selected based on the drug loading studies.

#### Preparation of Plain Ciprofloxacin Hydrochloride Gel

The ciprofloxacin hydrochloride gel was formulated for comparative studies with the developed nanocarriers loaded with ciprofloxacin hydrochloride. As there are no suitable marketed products available for comparative studies, a plain gel consisting of 1% *w*/*v* of Carbopol 974 P was formulated. Accurately weighed Carbopol 974 P was dispersed in distilled water and allowed to swell; the polymeric dispersion was kept constant by stirring for 60 min and neutralized using triethanolamine to obtain the clear gel. Ciprofloxacin hydrochloride weighing 500 mg was uniformly dispersed in a carbopol base. The developed gel was then evaluated for physicochemical parameters, and in vitro, drug release studies were used, as well as antimicrobial studies that were used for comparative data.

### 4.3. Identification Tests for Beta-Tricalcium Phosphate

#### 4.3.1. Identification Test for Phosphate

To a warm solution a slight excess of nitric acid ammonium molybdate was added and any color or precipitate formation was observed.

#### 4.3.2. Identification Test for Calcium

Beta tricalcium phosphate responded to the flame test for calcium.

About 100 mg of the sample was dissolved in 5 mL of dil. HCl and 5 mL of water by warming. Then, 1 mL of ammonia was added, dropwise with shaking, later 5 mL of ammonium oxalate was added and observed for any color or precipitate formation.

### 4.4. Characterization

#### 4.4.1. Micrometric Properties

Micromeritic properties such as angle of repose, Hausner ratio, and Carr’s index were performed to determine the flow properties of the developed nanocarriers.

##### Bulk Density

Bulk density is defined as the mass of particles of the material divided by the total volume they occupy. The total volume includes particle volume, inter-particle void volume, and internal pore volume. The maximum bulk density can be achieved without the deformation of the particles.

The required amount of powder was weighed and placed in a measuring cylinder and the volume occupied by it is noted.

The bulk density can be calculated by using the formula: $\frac{\mathrm{Mass}\;\mathrm{of}\;\mathrm{the}\;\mathrm{powder}}{\mathrm{Bulk}\;\mathrm{volume}}$.

##### Tapped Density

The tapped density is an increase in bulk density attained after mechanically tapping a container containing the powder sample.

The required amount of the powder was weighed and placed in the measuring cylinder and then it was tapped on a wooden base about 750–1250 times and the final volume occupied by it was noted.

The tapped density can be calculated by using the formula: $\frac{\mathrm{Mass}\;\mathrm{of}\;\mathrm{the}\;\mathrm{powder}}{\mathrm{Tapped}\;\mathrm{volume}}$.

##### Hausner’s Ratio

Hausner’s ratio is a number correlated to the flowability of a powder or a granular material. Hausner’s ratio is used in a wide range as an indication of the flowability of a powder.

The tapped density and bulk density were measured and the Hausner ratio was calculated using the formula: $\frac{\mathrm{Tapped}\;\mathrm{density}}{\mathrm{Bulk}\;\mathrm{density}}$.

A good flow is indicated by a Hausner’s ratio > 1.25 and a poor flow may have a value < 1.25.

##### Carr’s Compressibility Index

The Carr index also known as Carr’s index, or Carr’s compressibility index is an indication of the compressibility of a powder.

Carr’s index is frequently used as an indication of the flowability of a powder. In a free-flowing powder, the bulk density and the tapped density would be close in value; therefore, the Carr index would be small.

In a poorly flowing powder where there are greater interactions between particles, the observed difference between the bulk density and the tapped density would be greater, therefore the Carr index would be larger.

A Carr’s index greater than 25 is considered to be an indication of poor flowability and below 15 of good flowability. 

The bulk density and the tapped density were measured and the compressibility index was calculated using the formula: $\frac{\mathrm{Tapped}\;\mathrm{density}-\mathrm{Bulk}\;\mathrm{density}}{\mathrm{Tapped}\;\mathrm{density}}$ × 100.

##### Angle of Repose

The maximum angle possible between the surface and a powder pile of the horizontal plane is called the angle of repose.

The powders were allowed to flow through the funnel which is fixed to a stand at a definite height (h). The angle of repose was then calculated by measuring the granule height and radius of the heap formed. It is calculated by using the formula:

(1) $$Ø=\tan^{-1}\;\frac{h}{r}$$

where Ø = angle of repose, h = height of pile, and r = radius of the base of the pile.

#### 4.4.2. In Vitro Drug-Loading Studies

In vitro drug-loading studies were performed by taking different ratios of drug and carrier. The ratios were dissolved in suitable solvents, such as dilute hydrochloric acid, and settled overnight. The samples were filtered and estimated for drug concentration estimated using a UV–Visible spectrophotometer.

The amount of drug loaded was determined by the following formula:

(2) $$\%\;\mathrm{Drug}\;\mathrm{loading}=\frac{Ic-Fc}{Ic}\times 100$$

Ic = initial concentration, Fc = final concentration.

#### 4.4.3. Drug Content of Plain Gel

The drug content of plain gel loaded with ciprofloxacin hydrochloride was determined by diluting the gel equivalent to 100 mg of the drug [26]. The drug-loaded gel was diluted with distilled water and the mixture was vortexed for 10 min and subjected to centrifugation for 15 min at 1200 rpm. The supernatant solution was then analyzed for drug content using a UV-Visible spectrophotometer at 275nm.

#### 4.4.4. In Vitro Drug Release Studies

In vitro, drug release studies were performed in a glass vessel [26] by dispersing drug-loaded nanocarriers in 6.8 pH phosphate buffer. About 2 mL of supernatant liquid was removed for drug concentration estimation and replaced by fresh phosphate buffer at periodic intervals over a period of 5 days. Drug concentration was estimated in a UV–Visible spectrophotometer at 275 nm. Similar studies were carried out for the ciprofloxacin-loaded carbopol gel.

#### 4.4.5. Anti-Microbial Studies

Anti-microbial studies were carried out using the agar diffusion method for *Staphylococcus aureus* ATCC No. 6538 and *E. coli* ATCC No. 25922. The medium used for the antimicrobial study was Mueller–Hinton agar medium (Himedia Laboratories Pvt Ltd. Telangana, Telangana, India) which is most widely used for the antibiotic susceptibility test. The agar medium was prepared by dissolving 2.8 g of agar powder in 100 mL of distilled water and sterilized by using an autoclave at 121 °C for 15 min. The plates were first sterilized in a hot air oven at 160 °C for 60 min. After sterilization, the agar medium was poured into each Petri plate and solidified. The final concentration of microorganisms in the inoculums was [27] 108 cfu/mL and inoculated in the agar medium using a sterile inoculation loop. After solidification, a hole 6 mm in diameter was bored in the medium. In each plate, the equivalent dose was placed and incubated at 25 °C for 24 h. After, incubation zones with complete inhibition were measured and diameters of the same were recorded with the help of a measuring scale. The mean zone of inhibition was recorded for all the test samples and statistically analyzed using one-way ANOVA followed by Bonferroni’s multiple comparison tests at (* *p* < 0.05) significance level [26,27,28].

#### 4.4.6. Scanning Electron Microscopy (SEM Studies)

To evaluate the surface morphology of the nanocarriers and beta-tricalcium phosphate, the samples were examined by the FEI Quanta 200 ESEM scanning electron microscopy (SEM) model (FEI Company, Hillsboro, OR, USA). Prior to microscopy, samples were coated with gold/palladium by sputtering for 300 s in a bio rad. The samples were scanned at a voltage of 30 KV using scanning electron microscopy at 20.0 kx magnification.

#### 4.4.7. Differential Scanning Calorimetry (DSC)

DSC studies were performed to determine drug-excipient compatibility. The physical state of the ciprofloxacin hydrochloride, nanocarriers, and beta-tricalcium phosphate was characterized by using a differential scanning calorimeter (Mettler DSC 823e; Mettler-Toledo, Gießen, Germany) over a temperature range from 20 °C to 300 °C under a constant nitrogen gas flow of 30 mL/min at a heating rate of 10 °C/min. 

#### 4.4.8. Fourier Transform Infrared (FTIR) Spectroscopy

Infrared spectra of pure drug and physical mixture were recorded in a range of 3100 cm^−1^–800 cm^−1^ using an FTIR spectrophotometer (IR Prestige 21 Shimadzu model). FTIR was performed to identify functional groups and to understand the interactions between the drug and excipients.

#### 4.4.9. XRD Studies (XRD)

X-ray powder scattering measurements of ciprofloxacin hydrochloride, nanocarriers, and beta-tricalcium phosphate were carried out using an X’Pert PRO MPD diffractometer (PANalytical, Almelo, The Netherlands) with a copper anode (Cu Kα radiation = 0.15406 nm, 45 kV, 40 mA). The diffraction pattern was measured with a step size of 0.017 and a dwell time of 45 s at each step between 3 and 5° 2α at ambient temperature The powder X-ray diffraction patterns were recorded at room temperature. The XRD technique is used for the identification and characterization of compounds based on their diffraction patterns [25].

### 4.5. Statistical Analysis

Statistical analysis was performed using one-way analysis of variance (ANOVA) using GraphPad Prism software (Graph Pad Prism version 7.0.0.159, San Diego, CA, USA) [29,30]. A one-way analysis of variance (ANOVA) followed by Bonferroni’s multiple comparison tests was applied to determine the statistically significant difference in antibacterial activity between various formulations. The values obtained for formulations with a *p*-value of 0.05 were considered statistically significant. The characterization data were expressed as the means of three experiments ± SD.

## 5. Conclusions

Coral sand is a rich source of chemical constituents that are used due to their biocompatibility, osteoconduction, osteointegration, and osteogenetic effects. Coral sand is also used as a carrier for both immediate and sustained drug delivery formulations for various disease conditions. Coral has even been used as a substitute to treat various bone-related problems such as spinal fusion, fracture repairs, and filling of bone defects in periodontal and cranial-maxillofacial areas. β-tricalcium phosphate is one the most frequently used and potent synthetic bone graft substitutes that helps in osteogenesis and initiates cells to develop into preosteoblasts. Taking this into consideration, an attempt was made to extract β-tricalcium phosphate from coral sand and later convert it into nanocarriers by loading ciprofloxacin hydrochloride. The data obtained from the studies revealed that the ciprofloxacin hydrochloride-loaded nanocarrier (F1) released the drug for 120 h which was desired by the formulation to treat dental caries. The drug was released in controlled order for a prolonged period of time. The results of micrometric studies demonstrated that beta-tricalcium phosphate and nanocarriers had good and fair flowability. Formulation F1 consisting of a drug-to-carrier ratio of 1:1 showed the highest in vitro drug loading of up to 71.2% as compared to other formulations. Nanocarriers were found to be effective against *S. aureus* and *E. coli* with the greater zone of inhibition compared to control. The result of the FTIR and DSC studies reveals that there was no interaction between the drug and excipients. DSC studies also revealed that the drug was embedded in the matrix of β-TCP while XRD studies showed a marked decrease in the crystallinity of the drug in amorphous form. Since nanocarriers delivered the drugs to site-specific targets and released the drug in a controlled manner, they have a high potential to be used for treating dental caries.

## Figures and Tables

**Table 1:** Identification tests for beta tricalcium phosphate.

	Test	Observation	Inference
1	Test for phosphate	Yellow precipitate was observed	Yellow precipitate was formed
2	Flame test for calcium	Responds to flame test	Ash was formed
3	Test for calcium	White precipitate was observed	White precipitate was formed

**Table 2:** Micromeritics of nanocarriers formulation, an average of 3 determinations ± S.D.

Formulation Code	Angle of Repose (θ°) Mean ± SD *	Tapped Density (g/cm^3^) Mean ± SD *	Bulk Density (g/cm^3^) Mean ± SD *	Carr’s Index (%) Mean ± SD *	Hausner’s Ratio Mean ± SD *
F1	36 ± 1.8	0.78 ± 0.02	0.66 ± 0.09	15.55 ± 1.2	1.196 ± 0.25
F2	46 ± 0.89	0.96 ± 0.78	0.78 ± 0.98	15.59 ± 0.74	1.8 ± 0.85
F3	51 ± 0.76	0.84 ± 0.98	0.89 ± 0.66	37.09 ± 0.76	1.58 ± 0.34
F4	43 ± 0.45	0.54 ± 0.76	0.75 ± 0.90	28 ± 0.97	1.72 ± 0.56
F5	54 ± 0.78	0.53 ± 0.49	0.68 ± 0.81	45.01 ± 0.87	1.98 ± 0.71
F6	45 ± 0.69	0.95 ± 0.89	0.92 ± 0.29	26.86 ± 0.65	1.36 ± 0.69
F7	52 ± 0.97	0.82 ± 0.16	0.67 ± 0.99	30.09 ± 0.64	1.78 ± 0.6
F8	48 ± 0.5	0.73 ± 0.89	0.8 ± 0.56	18 ± 0.16	1.2 ± 0.4
F9	54 ± 0.32	0.79 ± 0.95	0.77 ± 0.78	17.01 ± 0.27	1.85 ± 0.13
F10	45 ± 0.31	0.88 ± 0.67	0.80 ± 0.18	26.86 ± 0.82	1.6 ± 0.93

* *p* < 0.05.

**Table 3:** Composition of nanocarriers’ formulation.

Formulation	Ratio (Drug and Carrier)	Drug (mg)	β-TCP (mg)	6.8 pH Phosphate Buffer Saline Solution (mL)
F1	1:1	500	500	75
F2	1:1	500	500	100
F3	1:1	500	500	150
F4	1:1	500	500	200
F5	1:2	125	250	75
F6	2:1	250	125	75
F7	1:3	125	375	75
F8	1:4	125	500	75
F9	3:1	375	125	75
F10	4:1	500	125	75

## Data Availability

Data is available with the investigators and may be provided upon reasonable request.
